# Utilization of maternal health services among young women in Kenya: Insights from the Kenya Demographic and Health Survey, 2003

**DOI:** 10.1186/1471-2393-11-1

**Published:** 2011-01-10

**Authors:** Rhoune Ochako, Jean-Christophe Fotso, Lawrence Ikamari, Anne Khasakhala

**Affiliations:** 1 African Population and Health Research Center (APHRC), P.O. Box 10787, 00100 Nairobi, Kenya; 2 Population Studies & Research Institute (PSRI), University of Nairobi. P.O. Box 30197, 00100 Nairobi, Kenya

## Abstract

**Background:**

Use of maternal health services is an effective means for reducing the risk of maternal morbidity and mortality, especially in places where the general health status of women is poor. This study was guided by the following objectives: 1) To determine the relationship between timing of first antenatal care (ANC) visit and type of delivery assistance 2) To establish the determinants of timing of first ANC visit and type delivery assistance.

**Methods:**

Data used were drawn from the 2003 Kenya Demographic and Health Survey, with a focus on young women aged 15-24. The dependent variables were: Timing of first ANC visit coded as *"None"; "Late" *and *"Early"*, and type of delivery assistance coded as *"None"*; "*Traditional Birth Attendant (TBA)" *and *"Skilled professional"*. Control variables included: education, household wealth, urban-rural residence, ethnicity, parity, age at birth of the last child and marital status. Multivariate ordered logistic regression model was used.

**Results:**

The study results show that place of residence, household wealth, education, ethnicity, parity, marital status and age at birth of the last child had strong influences on timing of first ANC visit and the type of delivery assistance received. The major finding is an association between early timing of the first ANC visit and use of skilled professionals at delivery.

**Conclusion:**

This study confirms that timing of first antenatal care is indeed an important entry point for delivery care as young women who initiated antenatal care early were more likely to use skilled professional assistance at delivery than their counterparts who initiated ANC late. The results indicate that a large percentage of young pregnant women do not seek ANC during their first trimester as is recommended by the WHO, which may affect the type of assistance they receive during delivery. It is important that programs aimed at improving maternal health include targeting young women, especially those from rural areas, with low levels of education, higher parity and from poor households, given their high risk during pregnancy. The finding that a considerably high proportion of young women use TBAs as opposed to use of skilled professionals is baffling and calls for further research.

## Background

Globally, a woman dies every minute from complications related to childbirth [1]. About half a million women die each year due to maternal causes with 99% of the deaths taking place in developing countries [1,2]. The challenge of reducing maternal mortality remains a major problem in Kenya. The 2003 Kenya Demographic and Health Survey (KDHS) estimated maternal mortality ratio (MMR) at 444/100,000 live-births. Other estimates put the ratio at 1,000/100,000 live births, representing a 1 in 25 lifetime risk of dying from a maternal-related cause [3,4]. Use of maternal health services is an effective approach to reducing the risk of maternal morbidity and mortality, especially in places where the general health status of women is poor [5-8]. Attending antenatal clinics and delivery with the assistance of skilled professionals (doctors and nurses) can lead to marked reductions in maternal morbidity and mortality through early detection and management of potential complications [5,6,9,10]. According to the World Health Organization (WHO), a maternal mortality is defined as the death of a woman while pregnant or within 42 days after the termination of a pregnancy, from any cause related to or aggravated by the pregnancy or its management but not from accidental causes [11,12].

Data from the Kenya Demographic and Health Survey (KDHS) show that although the overall ANC coverage remains high, many women make their first ANC visit late in pregnancy [4]. Importantly too, use of skilled professionals during delivery declined from 50% in 1989 to 42% in 2003, further demonstrating a deterioration in the use of maternal health services among women [4]. Key interventions through the Safe Motherhood initiative, the International Conference on Population and Development (ICPD) of 1994 and the 5^th ^Millennium Development Goal (MDG 5) have been adopted by the international community to improve maternal health by ensuring access to quality services in order to detect and manage life-threatening complications and reduce maternal morbidity and mortality [13-16].

According to the 2009 Kenya Preliminary Census report, young people (ages 15-24) who form about 36% of the total population, are the fastest growing segment of the population [17]. The young people face challenges of unemployment, early initiation into sex, abortions, unwanted pregnancies, and sexually transmitted diseases including HIV/AIDS among others [15]. For example, about 20% of the young women were either pregnant or had a birth at the time of the Kenya Demographic and Health Survey of 2003 [4]. Like many other health indicators, the burden of maternal morbidity and mortality is higher among this group as the risk of developing serious complications and subsequent death during pregnancy and childbirth are higher for them [16]. Furthermore, cultural and religious biases also discourage them from seeking reproductive health services, while some health providers are reluctant to provide contraceptives, to unmarried young women [18].

Many health providers have little training and experience in meeting special reproductive health needs of the young women and are ill equipped to solve their problems. This has contributed to a reduction in the use of maternal health services by the young women [19]. In addition, some married young women are disadvantaged in terms of decision making. For example, some have to consult their husbands and mothers-in-law before seeking maternal health care. These young women end up missing out on services geared towards adolescents because of their marital status, and are also denied services targeted towards married women because of their relatively young age, as well as lack of experience and autonomy [20-23].

Several studies have looked at the use of antenatal and delivery services, with a focus on the needs of women in the reproductive age [24-26]. This study is unique in that it focuses on timing of first ANC visit and type of delivery assistance sought by young women. Although frequency and timing of ANC visits are both important for timely identification and mitigation of potential pregnancy complications, this study only focused on timing of the first antenatal visit. This is because countrywide statistics indicate that use of antenatal care services in general is high, while initiation of antenatal visits is often delayed [4]. It is evident that the universally free ANC coverage in Kenya does not fully translate into use of skilled assistance since only 42% of births to all women are assisted by skilled professionals at the time of delivery. This study sought to investigate the association between the timing of young women's first ANC visit and their use of skilled professionals at delivery. The study results should contribute to our understanding of utilization of maternal health services and its associated determinants among the young women in Kenya. It is envisaged that a promptly executed first ANC visit would allow time for more such visits during the pregnancy, thereby enabling the woman to learn more about potential complications of pregnancy and the benefits that can be gained from professional delivery assistance. Such women are, therefore, better placed to seek professional assistance at delivery, hence the hypothesis that timely first ANC visit would be positively associated with professional delivery assistance.

The objectives of this study were;

i. To determine the linkage between timing of the first ANC visit and delivery assistance

ii. To establish the determinants of timing of first ANC visit and delivery assistance

## Methods

### Source of data

This study used data from the 2003 Kenya Demographic and Health Survey (KDHS), a publicly available and nationally representative survey of 8,195 women aged 15-49 years. The KDHS provides data on demographic and health indicators to promote studies on population, health, and nutrition of women and children in developing countries. The sample for the 2003 KDHS was drawn to allow for separate estimates of key indicators for each of the eight provinces in the country, and for urban and rural areas separately. As with other DHS surveys, the 2003 KDHS used a two-stage design: a) selection of clusters from a national master sample; and b) sampling of households from a list of all households in the sampled clusters [4]. This study used data on births that occurred in the three years preceding the survey period, and was restricted to the most recent births to 1675 young women aged 15-24 at the time of birth. Information on the most recent birth was used as it was assumed that the woman could vividly remember events during that pregnancy. The focus of the study was on young women aged 15-24 at the time of their last childbirth because this age bracket corresponds to the standard definition of young people as adopted and applied in many statistics and indicators by the United Nations, World Bank and the Government of Kenya [15,27]. There was no need for ethical clearance since the study involved secondary analysis of publicly available data.

### Study variables

There are two dependent variables both defined as three-category ordered variables: timing of first ANC visit and type of delivery assistance, derived from the following questions: *"How many months pregnant were you when you first received antenatal care for this pregnancy?" *and *"Who assisted with the delivery of (NAME OF CHILD)?" *The World Health Organization (WHO) recommends that for the majority of normal pregnancies, ANC should consist of at least four visits during the course of the pregnancy, the first of which should occur within the first trimester [28]. Timing of first ANC visit was thus recoded as *"None" *for those who did not receive ANC at all; *"Late" *when the visit took place during the second or third trimester, and *"Early" *when it occurred during the first trimester. Type of delivery assistance was recoded into three categories: *"None" *for no assistance, assistance from relatives or others without professional skills; "*TBA" *for assistance from traditional birth attendants (TBA); and *"Skilled professional" *for assistance from either a doctor or a nurse/midwife.

Based on previous studies on the use of maternal services, the independent variables used in this study include education (coded as none, primary and secondary/higher); household wealth; urban-rural residence; and ethnicity [9,25,29-32]. Since DHS do not collect data on income or expenditures, the economic status of household is proxied by a household wealth variable constructed from household possessions and amenities and dwelling characteristics, using principal component analysis [33]. For the purpose of this study, the resulting continuous variable (wealth index) was recoded as tertiles with categories labelled poor, middle and rich. Other control variables are parity, age at birth of the child and marital status.

### Methods of analysis

To achieve the first objective of the study, we used descriptive statistics on the timing of first ANC visit and type of delivery assistance. The statistics were weighted to adjust for differences in probability of selection and non-response. To establish the effects of the identified covariates on the two variables of interest (objective 2), bivariate and multivariate ordered logistic regression models were fitted in the context of the partial proportional odds model [34]. This model was chosen since the dependent variable is a three-category ordinal outcome. Using multinomial regression would mean that the information conveyed by the ordered nature of the outcome variable is discarded. In addition, not treating the variable as ordered, may lead to loss of efficiency [35]. The partial proportional odds model is a special case of the generalized ordered logit model that is less restrictive than the proportional odds model, which assumes that the hypothesis of parallel slopes is verified [34,36]. STATA command gologit2 was used to fit the partial proportional odds model [34].

## Results

### Sample description

Table 1 shows the characteristics of the sample of 1,675 women aged 15-24 at the birth of their last child. Nearly 70% of respondents were from rural areas; about two thirds had primary education and only 14% had no education. As expected, the sampled women were distributed almost equally in the three household wealth categories. With regard to ethnic affiliation, about 20% were Kikuyu, 18% were Luhya, 13% and 9% were Luo and Kamba, respectively. Half of the women had only one child, and slightly more than two thirds (68%) were aged 20-24. Three quarters of the women were in a union while the rest were either formerly or never married. Only 11% of the young women received their first ANC visit within the first trimester of pregnancy, 14% did not attend ANC in the course of their pregnancy. Table 1 also shows that half of the women received skilled professional assistance at delivery; a proportion slightly higher than that observed in the whole population of women aged 15-49 (42%). The proportion of births assisted by TBA was about 25% in our data, against 28% in the overall female population.

**Table 1:** Sample characteristics of women aged 15-24 at birth of last child

	Number	Percentage
**Place of residence**		
Urban	513	30.6
Rural	1,162	69.4
**Household wealth**		
Poor	630	37.6
Medium	551	32.9
Rich	494	29.5
**Education**		
None	235	14.0
Primary	1,087	64.9
Secondary+	353	21.1
**Ethnicity**		
Kikuyu	329	19.6
Luhya	293	17.5
Luo	222	13.3
Kamba	155	9.3
Other	676	40.4
**Parity (CEB)**		
1	845	50.5
2	488	29.1
3+	342	20.4
**Age at birth of last child**		
15-19	534	31.8
20-24	1,143	68.2
**Marital status**		
Never married	256	15.3
Formerly married	154	9.2
Currently married	1,265	75.5
**Timing of first ANC visit**		
None	227	13.6
Late	1,265	75.5
Early	183	10.9
**Type of delivery assistance**		
None	434	25.9
TBA	411	24.5
Skilled professional	830	49.6
**Total (n)**	**1,675**	**100.0**

### Timing of antenatal care (ANC)

Table 2 presents bivariate (Panel A) and multivariate (Panel B) analyses of timing of first ANC visit. The bivariate results show that place of residence was associated with timing of first ANC visit (p < 0.01), with rural women being less likely to go for ANC (Col. 1) or to make their first ANC visit during the first trimester of pregnancy (Col. 2). Multivariate coefficients for household wealth (Panel B) are in the expected direction although, surprisingly, only significant for middle household wealth (p < 0.10). These results are further emphasised in the bivariate model (Panel A) which indicates that women from middle income and rich households were more likely than their counterparts from poor households to go for ANC (p < 0.01) or to make their first visit in the first trimester (p < 0.05 in Col. 2).

**Table 2:** Odds ratio of various covariates on timing of first antenatal care visit among young women in Kenya

	**Panel A: Bivariate**				**Panel B: Multivariate**			
	
	**None vs (Late/Early)**		**(None/Late) vs Early**		**None vs (Late/Early)**		**(None/Late) vs Early**	
	
	Col. 1		Col. 2		Col. 3		Col. 4	
**Place of residence (Ref: Urban)**								
Rural	0.625	**	Same^a^		0.747		Same	
**Household wealth (Ref: Poor)**								
Medium	2.519	**	1.053		1.310	†	Same	
Rich	2.685	**	1.516	*	1.230		Same	
**Education (Ref: Primary)**								
None	0.200	**	1.716	**	0.244	**	1.947	**
Secondary+	1.323	†	Same		1.101		Same	
**Ethnicity (Ref: Kikuyu)**								
Luhya	0.615	*	Same		0.731		Same	
Luo	0.593	†	1.185		0.610	†	1.276	
Kamba	0.955		Same		1.057		Same	
Other	0.324	**	1.044		0.664	†	1.080	
**Parity (CEB) (Ref: 1)**								
2	0.824		Same		0.746	*	Same	
3+	0.368	**	0.663	†	0.455	**	Same	
**Age at birth of last child (Ref 15-19)**								
20-24	0.940		Same		1.119		Same	
**Marital status (Ref: Never married)**								
Formerly married	1.084		Same		1.515		Same	
Currently married	1.147		Same		1.936	**	Same	

^**†**^p < .10; ^*^p < .05; **p < .01

^**a**^Same: The Odds ratio for None vs (Late/Early) is the same as that of (None/Late) vs Early.

The relationship between education and early ANC visit appears counter intuitive in both the bivariate and multivariate models, with non-educated young women being more than 1.7 times more likely to seek ANC during the first trimester of pregnancy than their counterparts with primary education (p < 0.01). Similar results are confirmed in the multivariate model for women with no education. Bivariate results indicate that women with at least secondary education were more likely to go for ANC and make their first visit early (p < 0.10 in Panel A), compared to those with primary education. Kikuyu women were more likely to seek ANC compared to women from Luo and other ethnic groups. As expected, higher parity women were less likely to go for ANC and make the first visit during the first trimester, compared to those of parity one (Panel B). Currently married women were more likely to go for ANC and make their first visit during the first trimester (p < 0.01 in Panel B) compared to their never married counterparts.

### Type of delivery assistance

Results of bivariate (Panel A) and multivariate (Panel B) analyses on type of delivery assistance are as shown in Table 3. Rural women were less likely to deliver with the assistance of either TBA or skilled professional (p < 0.01 in both Panels). The effect of household wealth on delivery care appeared to be very strong, with women from rich households being more than three times more likely to deliver with TBA or skilled professional (p < 0.01 in Panel B) than those from poor households. These advantages are further emphasised in the bivariate model (Panel A) where women from rich households were more than four times more likely to deliver with TBA or skilled professionals (Col. 1) and 8 times more likely to deliver with skilled professional (Col. 2). The effect of education was strong in the expected direction with non-educated women less likely to deliver with assistance of skilled professional (p < 0.01 in Panel A) and surprisingly, more likely to deliver with TBA or skilled professionals (Col. 3) than those with primary education. Bivariate and multivariate models confirm that women with at least secondary education were 2.8 times more likely to deliver with TBA and or skilled professional (p < 0.01 in both Panels). Kikuyu women were more likely to deliver with TBA and or skilled professional compared to women from other ethnic groups - although their difference with Luhya women was not significant in the multivariate model (Col. 3). Interestingly, Luo women were more likely to deliver with any assistance (Col 3 and 4). Kikuyu women were better off in terms of delivery with skilled professional (Col. 2 and 4).

**Table 3:** Odds ratio of various covariates on type of delivery assistance among young women in Kenya

	**Panel A: Bivariate**				**Panel B: Multivariate**			
	
	**TrueNone vs (TBA/SPa)**		**True (None/TBA) vs SP**		**TrueNone vs (TBA/SP)**		**True (None/TBA) vs SP**	
	
	Col. 1		Col. 2		Col. 3		Col. 4	
**Place of residence (Ref: Urban)**								
Rural	0.942	**	0.264	**	0.791	**	0.561	**
**Household wealth (Ref: Poor)**								
Medium	1.400	**	2.726	**	1.157		1.540	**
Rich	4.285	**	8.256	**	3.033	**	3.033	
**Education (Ref: Primary)**								
None	0.978		0.243	**	1.602	**	0.359	**
Secondary+	2.864	**	Sameb		1.776	**	Same	
**Ethnicity (Ref: Kikuyu)**								
Luhya	0.627	*	0.165	**	0.890		0.200	**
Luo	1.402		0.307	**	1.681	*	0.284	**
Kamba	0.556	**	0.211	**	0.667	â€	0.218	**
Other	0.475	**	0.184	**	0.671	*	0.391	**
**Parity (CEB) (Ref: 1)**								
2	0.530	**	0.386	**	0.456	**	Same	
3+	0.419	**	0.219	**	0.378	**	Same	
**Age at birth of last child (Ref 15-19)**								
20-24	0.925		Same		1.600	**	1.130	
**Marital status (Ref: Never married)**								
Formerly married	1.821	**	Same		0.892		Same	
Currently married	1.792	**	Same		1.071		Same	
**Timing of ANC (Ref: None)**								
Late	1.338	†	4.133	**	1.123		2.053	**
Early	1.897	**	6.611	**	1.592	†	3.524	**

†p < .10; *p < .05; **p < .01

^**a**^SP: Skilled Professional

^**b**^Same: The odds ratio for None vs (TBA/SP) is the same as that of (None/TBA) vs SP.

Women who had more than two children were less likely to deliver with the assistance of TBA or skilled professionals (p < 0.01 in Panel B), compared with those with one child. The multivariate model shows that women aged 20-24 were more likely to deliver with TBA or skilled professionals (p < 0.01 in Col. 3). Bivariate results show that currently and formerly married women were more likely to deliver with TBA or and skilled professionals (p < 0.01 in Panel A).

**Figure 1:**
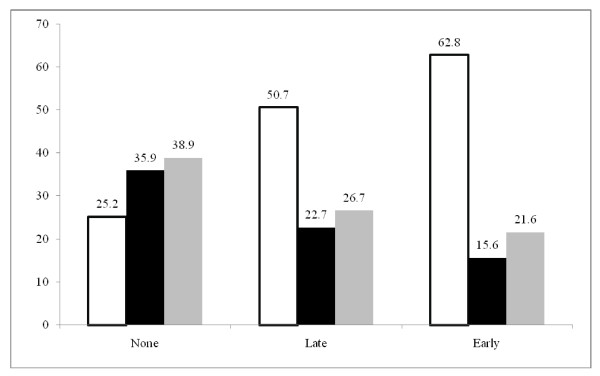
**Bivariate association between timing of ANC visit and type of delivery assistance**. □ Skilled professional ■ TBA ■ None.

Of special interest to the study is the extent to which the use of ANC services seems to affect the type of delivery assistance. As shown in Panel A of Table 3 (bivariate analysis), there is a graded relationship between use of ANC and delivery assistance, with women who went early for their first ANC visit being about twice as likely to deliver with the assistance of TBA or skilled professionals (p < 0.01 in Col. 1), and more than six times as likely to deliver with the assistance of a skilled professional (p < 0.01 in Col. 2), compared with their counterparts who did not go for ANC. Similarly, women who had their first ANC visit late were better off in terms of using skilled assistance at delivery than those who did not receive ANC. These results are further illustrated in Figure 1.

There is one noticeable limitation of the study. Because of the cross-sectional nature of the DHS data, it is not possible to draw robust conclusions on the effects of the identified covariates. For example, the dependent variable may have preceded a covariate if the index birth of a respondent occurred prior to the achievement of her current educational attainment. This limitation is inherent to DHS data. We have minimized this risk by limiting the dataset to births that occurred in the three years preceding the survey.

## Discussion

### Timing of antenatal care (ANC)

The purpose of the study was to explore utilization of maternal health care services among the young women in Kenya using data from the KDHS of 2003 [4]. Specifically, we investigated the link between timing of the first antenatal clinic visit and type of delivery assistance accessed by young women. Antenatal care is considered a key entry point for pregnant women to receive a broad range of health promotion and disease preventive services [37]. In this era of HIV/AIDS, it provides an opportunity for prevention of mother to child infection and promotes use of skilled assistance at delivery, which in turn promotes use of postpartum care services for the mother and child [6,38]. Despite the benefits of early timing of the first ANC visit, the majority of young women in Kenya delay to seek ANC. This could be due to their low social status and the fact that many of them have unplanned pregnancies [29,39].

Urban areas are usually characterised by better use of maternal health services, given their infrastructural advantages compared to rural areas. Results from this study confirm this advantage, but with no statistical significance. ANC is free in Kenya [4], therefore rural and urban women have an almost equal opportunity of getting the services thus explaining the observed weak relationship. Women from rich households were more likely to seek ANC early. Although cost is a barrier to seeking ANC especially among poor women who cannot access free government health facilities, rich women have other advantages such as access to information about the benefits of early initiation of care. This association has been confirmed elsewhere where low socio-economic status and under-utilization of maternal health services were found to be interlinked [30,38].

Women with primary education were less likely to receive ANC in the first trimester. The first possible explanation is the fact that the proportion of women with no education (14%) is low compared to that of women with primary education (6roportion of women with no education (14%) is low compared to those with primary education (145%). Less education is associated with increased chances of early marriage, which is likely to provide access to health care services geared towards married women. In their study, Bicego et. al. (1993) found that women with less education have a reduced risk of neonatal mortality due to benefits associated with family formations [40,41]. The effect of ethnic variation was evident with Kikuyu women being more likely to receive ANC in the first trimester compared to women from other ethnic groups. This ethnic advantage could be due to traditional beliefs and cultural practices specific to the different groups and the proximity of Kikuyu women to Nairobi, where it is assumed that they benefit from the flow of information on the benefits of ANC [30].

This study confirms findings in other studies that higher parity women are less likely to initiate ANC or make the first visit in the first trimester [30,40]. Women aged 20-24 were more likely to initiate ANC and make their first visit in the first trimester although this difference was not statistically significant. This could be due to the fact that there are no major differences between women aged 15-19 and 20-24. Women who were currently in marriage were more likely to receive the first ANC in the first trimester. This advantage could be due to the support married women receive from husbands, and the health delivery system that tends to favour married women over unmarried young women [42].

### Type of delivery assistance

Our findings reveal that 50% of the young women use skilled professional assistance at delivery. This is slightly more than the national average of 42% for all women of reproductive age [4]. This finding supports those of several studies which confirmed that younger women beginning child bearing tend to fear home deliveries as they consider themselves a high risk group. As a result, such young women seek professional assistance from skilled professionals in hospitals [5,39]. On the contrary, a study by Magadi et al (1999) found teenagers to be more likely to deliver at home [31], although the difference in these results could be due to the different age groups examined by the two studies. Considering the higher risks young women face during pregnancy and childbirth, the results of this study should not be interpreted to mean that young women are better users of skilled professional assistance [6,43]. It is estimated that maternal deaths during birth are 2-4 times higher among young women, and babies born to them have a higher risk of death during the neonatal period due to low birth weight, compared to older women [44].

A number of socio-economic and demographic factors had significant influence on use of skilled professional assistance at delivery. These include place of residence, household wealth, education, ethnicity, parity and marital status. Urban young women were more likely to use skilled professional assistance compared to rural young women. Some advantages urban women have over their rural counterparts are higher levels of knowledge, access to services and health promotion programs that use urban-focused mass media, thus leaving out their rural counterparts who may be largely influenced by traditional practices [45]. As expected, educated young women were better users of skilled professional assistance. This is consistent with findings elsewhere [29,30,39,45]. Educated and single women have higher autonomy to make decisions on the quality of health care they receive [5].

As reported in other studies, higher parity women were less likely to use skilled professional assistance at delivery, as they feel experienced and knowledgeable from previous birth experiences [45]. Young women from rich households were more likely to use skilled professional assistance at delivery while Kikuyu women were more likely to be users of skilled professionals at delivery. This advantage of the Kikuyu women over other ethnic groups has been attributed to superior access to services and flow of information from urban areas given their proximity to Nairobi city [4]. Never married women are assumed to have higher autonomy and do not depend on decisions of others, unlike their married counterparts who depend on decisions of their husbands and mothers-in-law. Married young women are also more likely to get assistance from relatives at home during delivery as opposed to their unmarried counterparts. There was a strong association between early timing of first ANC visit and use of skilled professional assistance at delivery, thereby confirming the notion that antenatal could be an important entry point for delivery care as women who sought antenatal care early were more likely to use skilled professional assistance at delivery [37].

## Conclusion

This study confirms that timing of first antenatal care is indeed an important entry point for delivery care as women who initiated antenatal care early were more likely to use skilled professional assistance at delivery than their counterparts who initiated ANC late. The results indicate that a large percentage of young pregnant women do not seek ANC during their first trimester as is recommended by the WHO, which may affect the type of assistance they receive during delivery. It is important that programs aimed at improving maternal health include targeting young women, especially those from rural areas, with low levels of education, higher parity and from poor households, given their high risk during pregnancy. The finding that a considerably high proportion of young women use TBAs as opposed to use of skilled professionals is baffling and calls for further research.

## Competing interests

The authors declare that they have no competing interests.

## Authors' contributions

RO: Participated in the origination of the research idea and played lead roles in conducting literature review, data analysis, writing the results and discussion sections. JCF: Participated in data analysis and writing the results section. LI: Participated in writing the introduction section. AK: Participated in writing the discussion section. All authors read and approved the final manuscript.

## Pre-publication history

The pre-publication history for this paper can be accessed here:

http://www.biomedcentral.com/1471-2393/11/1/prepub
